# Src Family Tyrosine Kinase Signaling Regulates FilGAP through Association with RBM10

**DOI:** 10.1371/journal.pone.0146593

**Published:** 2016-01-11

**Authors:** Hazuki Yamada, Koji Tsutsumi, Yuki Nakazawa, Yoshio Shibagaki, Seisuke Hattori, Yasutaka Ohta

**Affiliations:** 1 Division of Cell Biology, Department of Biosciences, School of Science, Kitasato University, Kanagawa, 252–0373, Japan; 2 Division of Biochemistry, School of Pharmaceutical Sciences, Kitasato University, Tokyo, 108–8641, Japan; University of Toledo, UNITED STATES

## Abstract

FilGAP is a Rac-specific GTPase-activating protein (GAP) that suppresses lamellae formation. In this study, we have identified RBM10 (RNA Binding Motif domain protein 10) as a FilGAP-interacting protein. Although RBM10 is mostly localized in the nuclei in human melanoma A7 cells, forced expression of Src family tyrosine kinase Fyn induced translocation of RBM10 from nucleus into cell peripheries where RBM10 and FilGAP are co-localized. The translocation of RBM10 from nucleus appears to require catalytic activity of Fyn since kinase-negative Fyn mutant failed to induce translocation of RBM10 in A7 cells. When human breast carcinoma MDA-MB-231 cells are spreading on collagen-coated coverslips, endogenous FilGAP and RBM10 were localized at the cell periphery with tyrosine-phosphorylated proteins. RBM10 appears to be responsible for targeting FilGAP at the cell periphery because depletion of RBM10 by siRNA abrogated peripheral localization of FilGAP during cell spreading. Association of RBM10 with FilGAP may stimulate RacGAP activity of FilGAP. First, forced expression of RBM10 suppressed FilGAP-mediated cell spreading on collagen. Conversely, depletion of endogenous RBM10 by siRNA abolished FilGAP-mediated suppression of cell spreading on collagen. Second, FilGAP suppressed formation of membrane ruffles induced by Fyn and instead produced spiky cell protrusions at the cell periphery. This protrusive structure was also induced by depletion of Rac, suggesting that the formation of protrusions may be due to suppression of Rac by FilGAP. We found that depletion of RBM10 markedly reduced the formation of protrusions in cells transfected with Fyn and FilGAP. Finally, depletion of RBM10 blocked FilGAP-mediated suppression of ruffle formation induced by EGF. Taken together, these results suggest that Src family tyrosine kinase signaling may regulate FilGAP through association with RBM10.

## Introduction

Rho family small GTPases (Rho GTPases) regulate multiple cellular behaviors such as cell migration, invasion, spreading, and adhesion. They are involved in signaling downstream of cell-matrix adhesion, leading to control of actin cytoskeleton and cell migration [1–5]. Rho GTPases function as molecular switches in cells. They cycle between active GTP–bound and inactive GDP-bound forms. This cycle is mainly regulated by two classes of proteins. Guanine nucleotide exchange factors (GEFs) activate Rho GTPases by loading GTP, whereas GTPase-activating proteins (GAPs) facilitate the inactivation of Rho GTPases by stimulating their intrinsic GTPase activity [1–7].

FilGAP is a Rac-specific GTPase-activating protein that suppresses Rac-dependent cell spreading, migration, and lamellae formation [8–17]. Phosphorylation of FilGAP by Rho/ROCK stimulated RacGAP activity [8]. Forced expression of FilGAP induced membrane blebbing and ROCK inhibitor suppressed bleb formation. Conversely, depletion of endogenous FilGAP by siRNA stimulated lamellae formation. Thus, FilGAP mediates antagonism of Rac by Rho, which suppresses lamellae formation and promotes cell contraction [14,15,18,19]. FilGAP binds to actin-filament crosslinking protein filamin A and suppresses integrin-mediated cell spreading on fibronectin [8]. A FilGAP isoform lacking PH domain (RC-GAP) is associated with focal adhesion [20].

RBM10 (RNA Binding Motif domain protein 10) is an RNA-binding protein and regulates alternative splicing [21–23]. RBM10 contains two RNA recognition motifs (RRM), two zinc fingers (ZF) together with an octamer-repeat region and a G-patch domain [24,25]. Previous studies have demonstrated that RBM10 is frequently mutated in lung adenocarcinoma [26,27], and associated with TARP (talipes equinovarus, atrial septal defect, Robin sequence, and persistent left superior vena cava) syndrome [28]. RBM10 is directly tyrosine-phosphorylated by c-Src, a member of Src family tyrosine kinases [29]. However, it is unclear how RBM10 is regulated downstream of Src kinase signaling.

Src is a member of a family of non-receptor cytoplasmic tyrosine kinases, which becomes activated following stimulation of plasma membrane receptors and integrins [30]. Src family kinases (Src, Fyn, and Yes) are ubiquitously expressed in various tissues and involved in the regulation of diverse cellular functions including cell proliferation, survival, adhesion, and cell migration. Integrin-mediated cell adhesion stimulates Src family kinases and induces cell migration by modulating activity of Rho small GTPases [31,32]. RhoGEFs (such as VAV and Tiam1) and RhoGAPs (such as p190RhoGAP) are activated by Src-dependent phosphorylation [31,32]. Src family kinases also induce recruitment and phosphorylation of adaptor proteins, which in turn recruit and activate RacGEFs such as DOCK180 and ßPIX [31,32]. Src family kinases regulate Rho GTPases by GEFs and GAPs. It has been shown that cell spreading on extracellular matrix (ECM) induces up- and down-regulation of Rac and Rho through activation and inactivation of RhoGAPs and RhoGEFs [18]. Growth factors such as EGF also stimulate Src family tyrosine kinases and regulate Rho GTPases [30,33,34].

In this study, we identified RBM10 as a FilGAP-interacting protein in mammalian cells. We present evidence that Src family tyrosine kinase signaling may regulate localization and RacGAP activity of FilGAP through association with RBM10.

## Results

### FilGAP interacts with RBM10 in mammalian cells

**Fig 1:**
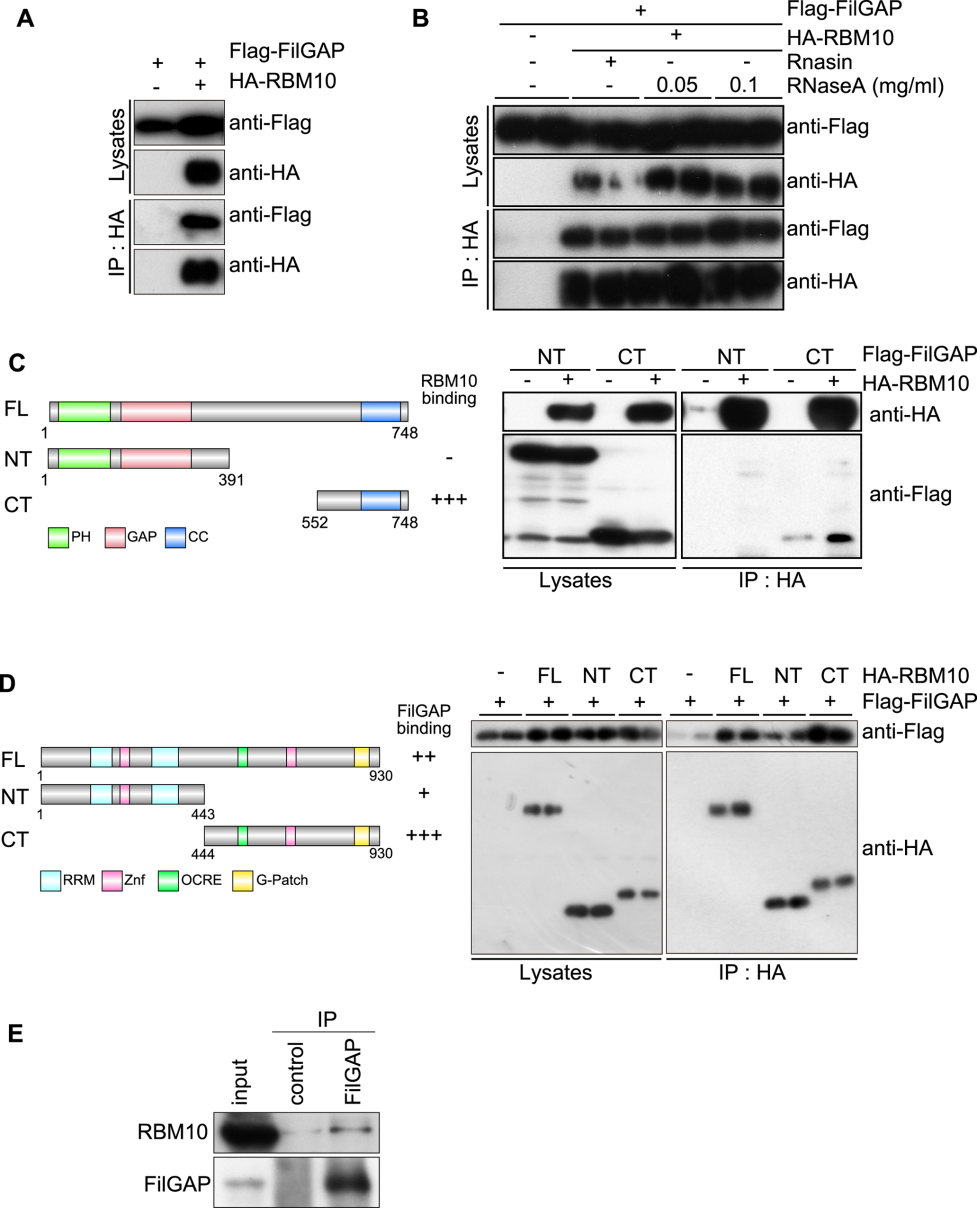
Binding of FilGAP to RBM10. (**A**) Co-precipitation of FilGAP and RBM10. HEK293T cells were transiently transfected with HA-tagged RBM10 (HA-RBM10) and Flag-tagged FilGAP (Flag-FilGAP). Cell extracts were prepared and HA-RBM10 was immunoprecipitated using an anti-HA antibody. The washed immunoprecipitates were immunoblotted for the presence of FilGAP and RBM10. (**B**) HEK293T cells were transiently transfected with HA-RBM10 and Flag-FilGAP. Cell extracts were prepared and incubated without or with RNase inhibitor Rnasin (0.12 mg/ml), RNase A (0.05 mg/ml), or RNase A (0.1 mg/ml) for 20 min at 37°C. Then, HA-RBM10 was immunoprecipitated using an anti-HA antibody. The washed immunoprecipitates were immunoblotted for the presence of FilGAP and RBM10. Two separate samples are shown. (**C**) HEK293T cells were transiently transfected with HA-RBM10 and Flag-FilGAP constructs shown in the diagram. Cell extracts were prepared and HA-RBM10 was immunoprecipitated using an anti-HA antibody. The washed immunoprecipitates were immunoblotted for the presence of RBM10 and FilGAP. Two separate samples are shown. PH; Pleckstrin homology, GAP; Rho GAP, CC; coiled-coil domain (**D**) HEK293T cells were transiently transfected with Flag-FilGAP and HA-RBM10 constructs shown in the diagram. Cell extracts were prepared and HA-RBM10 was immunoprecipitated using an anti-HA antibody. The washed immunoprecipitates were immunoblotted for the presence of RBM10 and FilGAP. Two separate samples are shown. RRM1; RNA recognition motif 1, Zf; Zink finger; OCRE; octamer-repeat region, G-patch; G-patch domain (E) Endogenous FilGAP associates with endogenous RBM10. MDA-MB-231 cells were incubated with 1 mM dithiobis (succinimidyl propionate) at 25°C for 10 min, washed with PBS and suspended in lysis buffer. Cell lysates were prepared and FilGAP protein was immunoprecipitated using anti-FilGAP antibody and bound RBM10 was identified using anti-RBM10 antibody.

To find binding partners of FilGAP, we performed immunoprecipitation with anti-Flag M2 antibody using HEK293T cells over-expressing Flag-tagged C-terminal fragment of FilGAP protein (a.a. 373–748). Co-immunoprecipitated proteins were isolated and analyzed by LC-MS/MS. RBM10 was identified as a binding candidate. To confirm binding, full-length Flag-FilGAP and HA-RBM10 were transiently transfected into HEK293T cells. HA-RBM10 was immunoprecipitated from cell lysates. As shown in Fig 1A, Flag-FilGAP was co-precipitated with HA-RBM10.

RBM10 is an RNA-binding protein and many RNA-binding proteins are known to be present as a large molecular complex with RNA [35,36]. Therefore, we next examined if RNA is involved in the interaction between FilGAP and RBM10. Treatment of the cell extracts with RNase or RNase inhibitor (Rnasin) did not affect the co-precipitation of RBM10 and FilGAP (Fig 1B). Thus, RNA does not seem to be involved in the interaction between FilGAP and RBM10.

We delineated respective binding sites between FilGAP and RBM10. To identify the binding domain in FilGAP, HEK293T cells were transfected with full-length HA-RBM10 and Flag-FilGAP fragments (Fig 1C). HA-RBM10 protein was precipitated from the cell lysates and co-precipitated Flag-FilGAP fragments were examined by Western blot. Flag-tagged C-terminal of FilGAP (CT), but not N-terminal half of Flag-FilGAP (NT), was co-precipitated with HA-RBM10 (Fig 1C).

Next, we determined binding sites in RBM10. HEK293T cells were transfected with full-length Flag-FilGAP and HA-RBM10 fragments (Fig 1D). HA-RBM10 protein was precipitated from the cell lysates and co-precipitated Flag-FilGAP was examined by Western blot. Compared to N-terminal half of HA-RBM10 (NT), significant amount of Flag-FilGAP was co-precipitated with C-terminal half of HA-RBM10 (CT). Taken together, these results suggest that C-terminal half of RBM10 mainly interacts with C-terminal half of FilGAP. To determine if endogenous RBM10 could interact with endogenous FilGAP, we studied MDA-MB-231 cells because the cells abundantly express endogenous FilGAP and RBM10 proteins. MDA-MB-231 cells were treated with cross-linker dithiobis (succinimidyl propionate) and cell lysates were prepared. Anti-FilGAP antibodies precipitated endogenous RBM10 with endogenous FilGAP from the cell lysates (Fig 1E).

### Fyn regulates localization of RBM10 and FilGAP

**Fig 2:**
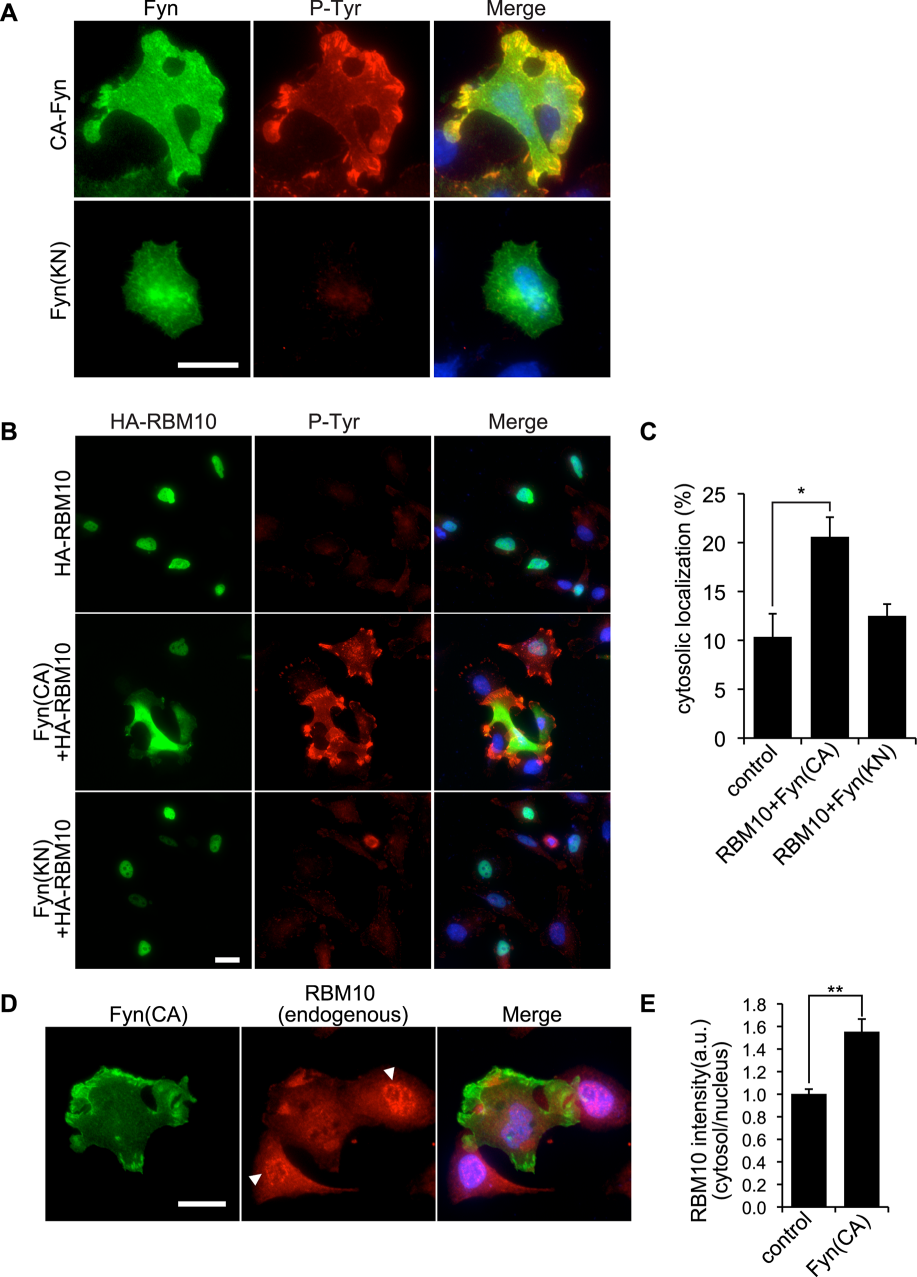
Fyn regulates localization of RBM10 in A7 cells. (**A**) A7 cells were transfected with constitutively activated Fyn (CA-Fyn) or kinase negative Fyn (KN-Fyn) and serum starved. After 24 h, cells were fixed and Fyn (green) and tyrosine-phosphorylated proteins (red) were localized by staining the cells with anti-Fyn and anti-pTyr antibodies. Merged fluorescent images are shown. Scale bar, 25 μm. Cells were also stained with Hoechst 33258 to label nuclei (blue). (**B**) A7 cells were transfected with HA-RBM10 together with CA-Fyn or KN-Fyn and serum starved. After 24 h, cells were fixed and HA-RBM10 (green) and tyrosine-phosphorylated proteins (red) were localized by staining the cells with anti-HA and anti-pTyr antibodies. Merged fluorescent images are shown. Cells were also stained with Hoechst 33258 to label nuclei (blue). Scale bar, 25 μm. (**C**) The percentages of cytosolic RBM10 were calculated and the data are expressed as the mean +/- s.e.m. (n = 3–6). *, *p*<0.05. (**D**) A7 cells were transfected with CA-Fyn and serum starved. After 24 h, cells were fixed and CA-Fyn (green) and endogenous RBM10 (red) were localized by staining the cells with anti-Fyn and anti-RBM10 antibodies. Merged fluorescent images are shown. Scale bar, 25 μm. Arrowheads indicate the nuclear localization of RBM10 in control cells. (E) The ratio of cytosolic and nuclear RBM10 fluorescence intensities are shown (n = 20 cells). The data are expressed as the mean +/- s.e.m. **, *p*<0.01.

A previous study has reported that RBM10 is directly tyrosine-phosphorylated by c-Src [29]. Therefore, we determined if localization of RBM10 is affected by Src signaling. To induce Src signaling, we used constitutively activated Fyn mutant (CA-Fyn), which is a member of Src family tyrosine kinases. Expression of CA-Fyn, but not kinase-negative Fyn mutant (KN-Fyn), in human melanoma A7 cells induced tyrosine phosphorylation (Fig 2A).

When A7 cells were transfected with HA-RBM10, it was mostly localized in the nucleus (Fig 2B and 2C). Co-expression of CA-Fyn with HA-RBM10 induced translocation of HA-RBM10 from nucleus to cell peripheries (Fig 2B and 2C). On the other hand, co-expression of kinase-negative KN-Fyn and HA-RBM10 failed to induce translocation of HA-RBM10 from nucleus. This may suggest that translocation of RBM10 from nucleus requires kinase-activity of Fyn.

**Fig 3:**
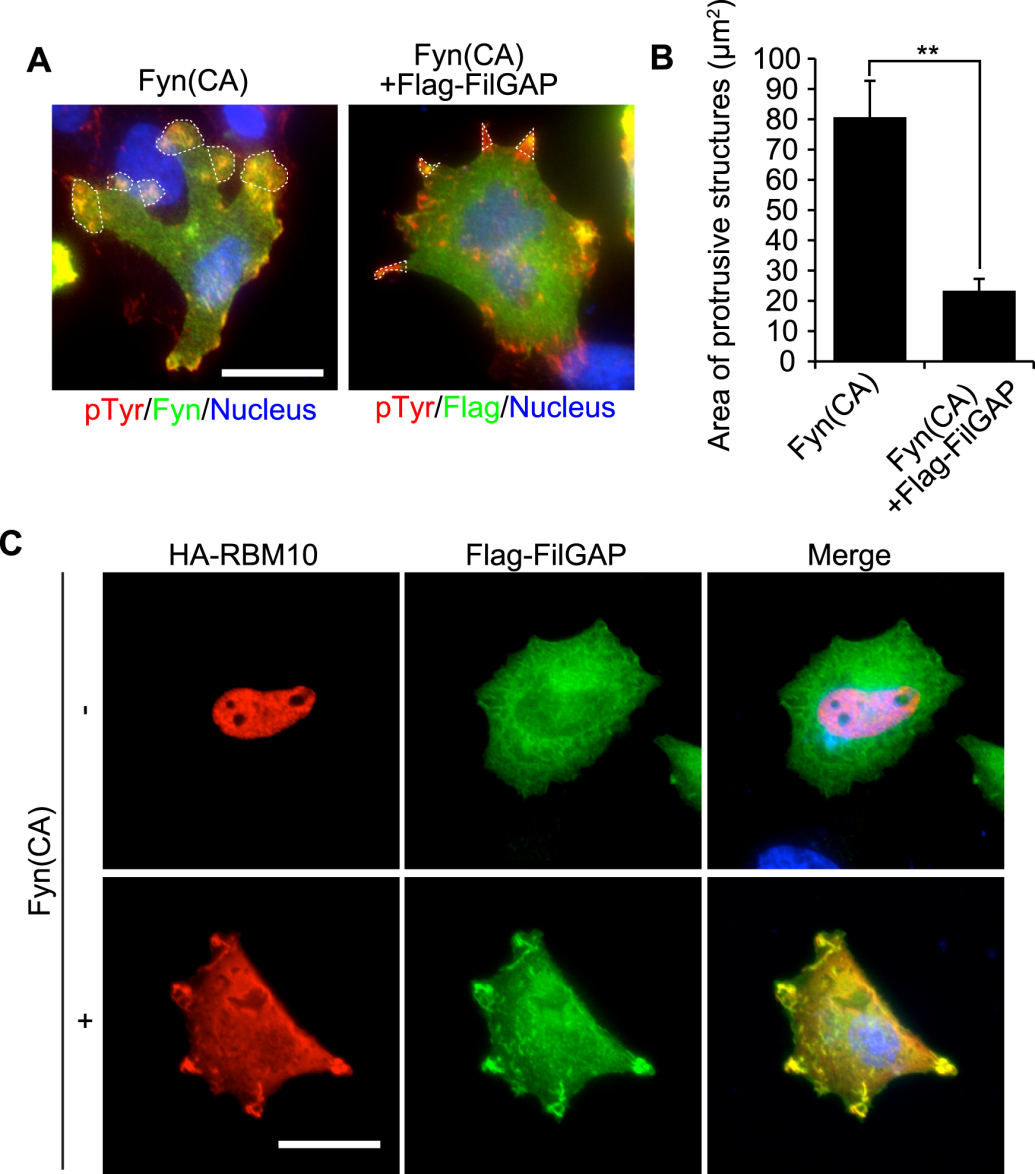
Fyn regulates localization of RBM10 and FilGAP in A7 cells. (**A**) A7 cells were transfected with CA-Fyn in the absence or presence of Flag-FilGAP and serum starved. After 24h, cells were fixed and stained with anti-Fyn (green) or anti-Flag (green), and anti-pTyr (red) antibodies. Cells were also stained with Hoechst 33258 to label nuclei (blue). Merged fluorescent images were shown. Characteristic protrusive structures were indicated with dotted lines. Scale bar, 25 μm. (**B**) The surface area of protrusive structures as indicated by dotted lines in (**A**) was calculated (n = 15–20 cells), and the data are expressed as the mean +/- s.e.m. **, *p*<0.01. (**C**) A7 cells were transfected with HA-RBM10 and Flag-FilGAP in the absence or presence of CA-Fyn and serum starved. After 24h, cells were fixed and HA-RBM10 (red) and Flag-FilGAP (green) were localized by staining the cells with anti-HA and anti-Flag antibodies. Cells were also stained with Hoechst 33258 to label nuclei (blue). Merged fluorescent images were shown. Scale bar, 25 μm.

Over-expression of CA-Fyn in A7 cells induced large lamellae at the cell periphery (Fig 3A). We found forced expression of FilGAP suppressed lamellae formations induced by CA-Fyn and instead produced small cell protrusions at the cell periphery (Fig 3A and 3B). In the absence of CA-Fyn, most of RBM-10 is localized in the nucleus and co-localization of FilGAP is not observed by immunofluorescent staining (Fig 3C). However, transfection of A7 cells with HA-RBM10 and Flag-FilGAP in the presence of CA-Fyn induced co-localization of Flag-FilGAP and HA-RBM10 at small cell protrusions at the cell periphery (Fig 3C). The findings suggest that localization of FilGAP and RBM10 may be regulated downstream of Src family tyrosine kinases.

### RBM10 targets FilGAP to tyrosine-phosphorylated proteins at the cell periphery

**Fig 4:**
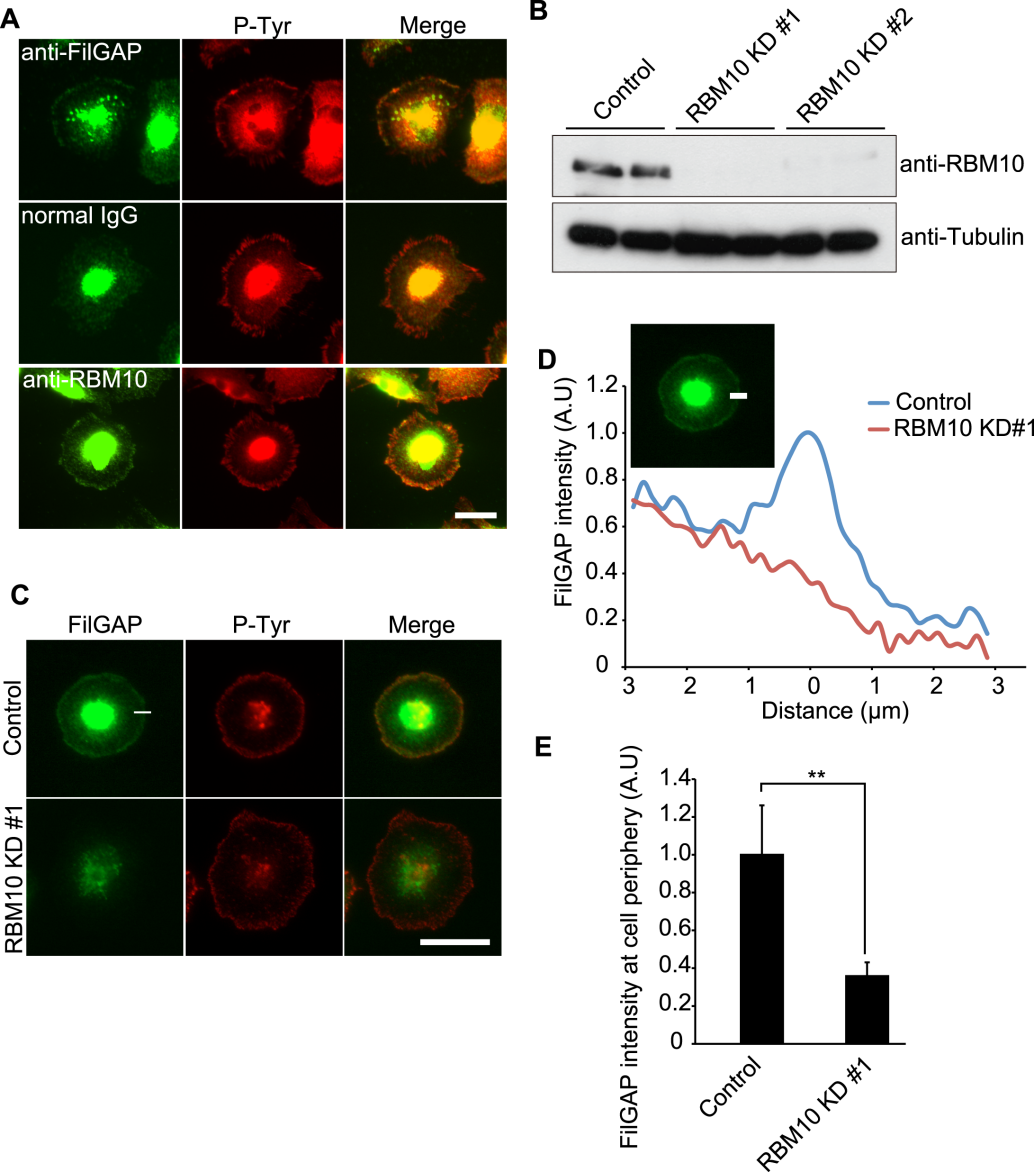
Localization of endogenous FilGAP and RBM10 in MDA-MB-231 cells spreading on collagen. (**A**) MDA-MB-231 cells were serum-starved for 18 hrs. Quiescent cells were trypsinized and cells in suspension were plated on coverslips coated with collagen and fixed at 30 min after plating. Cells were stained with control IgG, anti-FilGAP, or anti-RBM10 antibodies for endogenous FilGAP and RBM10 proteins (green). Cells were also stained with anti-pTyr antibody for visualization of focal complex (red). Merged fluorescent images are shown. Scale bar, 25 μm. (**B**) Immunoblot showing that RBM10 is depleted after 48hr of siRNAs treatment. Tubulin was used as a loading control. (**C**) MDA-MD-231 cells were transfected with control or RBM10 siRNA. After 30 hrs, the cells were serum-starved for 18 hrs and trypsinized, and cells in suspension were plated on coverslips coated with collagen and fixed at 30 min after plating. Cells were stained with anti-FilGAP antibody (green) for endogenous FilGAP, and anti-phosphotyrosine antibody (red) for tyrosine-phosphorylated proteins. Merged fluorescent images are also shown. Scale bar, 25 μm. (**D**) Fluorescent intensities of endogenous FilGAP were quantified by line scan analysis. (**E**) Fluorescent intensities of endogenous FilGAP at the cell periphery are shown, and the data are expressed as the mean +/- s.e.m. **, *p*<0.01. The statistical significance was accessed by Student’s t-test. (control: n = 11, RBM10KD: n = 9)

When serum-deprived quiescent cells are spreading on collagen, ligation of integrin stimulates tyrosine-kinase signaling and tyrosine phosphorylated proteins are clustered as small focal complexes around cell peripheries (Fig 4A). Because forced expression of CA-Fyn induced co-localization of transfected FilGAP and RBM10 at the cell periphery (Fig 3C), we next examined if endogenous FilGAP and RBM10 could be co-localized at focal complex when cells are spreading on collagen. When MDA-MB-231 cells were plated on collagen-coated dishes for 30 min, endogenous FilGAP and RBM10 were localized with tyrosine-phosphorylated proteins at the cell periphery as detected by anti-FilGAP and anti-RBM10 antibodies (Fig 4A). The peripheral localization of FilGAP and RBM10 seems to be specific because the peripheral staining was not detected by normal IgG (Fig 4A). To study the role of endogenous RBM10 in the regulation of FilGAP, we have transfected cells with small interference RNAs (siRNA) targeting RBM10. Two independent siRNAs targeting RBM10 (KD#1 and KD#2) reduced the expression of endogenous RBM10 (Fig 4B). We found that depletion of RBM10 by siRNA abrogated localization of FilGAP with tyrosine-phosphorylated proteins at cell peripheries (Fig 4C–4E). Therefore, RBM10 seems to be required for targeting of FilGAP at the cell periphery when cells are spreading on collagen.

### RBM10 promotes suppression of integrin-mediated cell spreading by FilGAP

**Fig 5:**
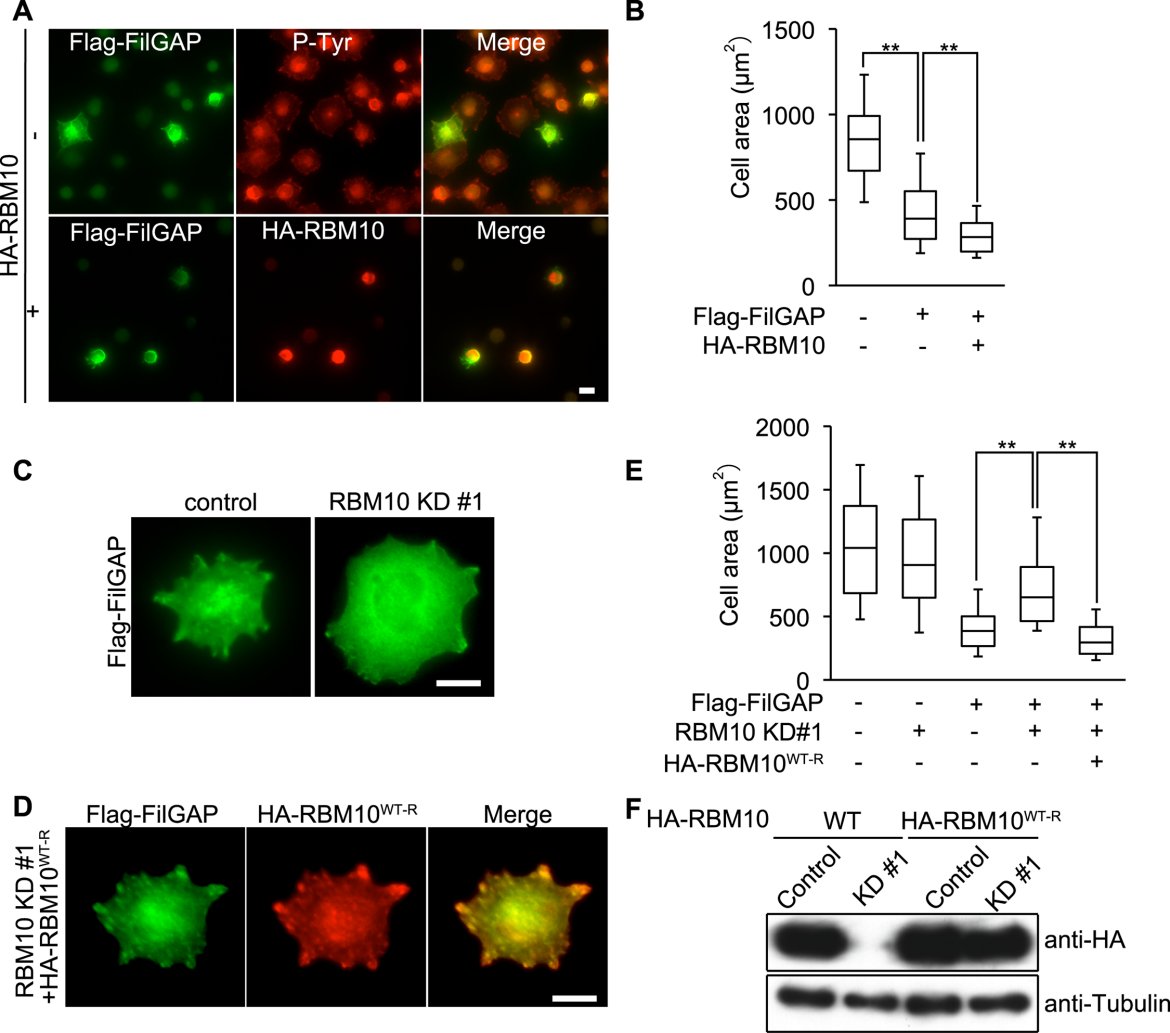
RBM10 promotes FilGAP-dependent suppression of cell spreading on collagen. (**A**) A7 cells were transiently transfected with Flag-FilGAP without (-) or with (+) HA-RBM10 and serum starved. Quiescent cells were trypsinized, and then plated on coverslips coated with collagen and fixed at 30 min after plating. Cells were fixed and stained with anti-FilGAP (green) and anti-pTyr (red) or anti-HA (red) antibodies. Merged fluorescent images are also shown. Scale bar, 25 μm. (**B**) The surface area of spreading cells 30 min after plating was calculated and the data are expressed as box plots. The box represents the 25–75th percentiles, and the median is indicated. The whiskers show the10–90th percentiles. **, *p*<0.01. The statistical significance was determined by Welch’s *t*-test. (n = 150 cells) (**C**) A7 cells were transiently transfected with Flag-FilGAP and in the presence of control or RBM10 siRNAs and serum starved. Quiescent cells were trypsinized, and then plated on coverslips coated with collagen and fixed at 30 min after plating. Cells were stained with anti-FilGAP (green) antibody. Scale bar, 25 μm. (**D**) A7 cells were transiently transfected with Flag-FilGAP and HA-RBM10 resistant to RBM10 siRNA (HA-RBM10^WT-R^) in the presence of RBM10 siRNA and serum starved. Quiescent cells were trypsinized, and then plated on coverslips coated with collagen and fixed at 30 min after plating. Cells were stained with anti-FilGAP (green) and anti-HA (red) antibodies. Merged fluorescent images are also shown. Scale bar, 25 μm. (**E**) The surface area of spreading cells 30 min after plating was calculated and the data are expressed as box plots. The box represents the 25–75th percentiles, and the median is indicated. The whiskers show the10–90th percentiles. **, *p*<0.01. The statistical significance was determined by Welch’s *t*-test. (n = 150–300 cells) (**F**) HEK293T cells were transiently transfected with HA-RBM10 or HA-RBM10^WT-R^ in the presence of control or RBM10 siRNA. HA-RBM10 proteins were analyzed by immunoblot using anti-HA antibody. Tubulin was used as a loading control.

Integrins link ECM components to the actin cytoskeleton, and interact with multiple structural and signaling molecules inside the cells including focal adhesion kinase (FAK) and Src [37,38]. Activation of FAK/Src tyrosine kinases controls cell adhesion and migration through regulation of Rho GTPases [39]. We have shown previously that FilGAP suppressed Rac activity and abolished integrin-mediated cell spreading [8]. Moreover, our present study showed that targeting of FilGAP to focal complexes at cell periphery is dependent on RBM10 during cell spreading on collagen (Fig 4C). Therefore, we examined if RBM10 is involved in the regulation of FilGAP activity downstream of integrin (Fig 5). Human melanoma A7 cells that were plated on collagen-coated dishes adhered and then spread by 1h. Over-expression of FilGAP abolished cell-spreading and the spread area that were occupied by FilGAP-transfected cells is smaller than that of control cells 30 min after spreading (Fig 5A and 5B). We found that transfection of RBM10 together with FilGAP augmented FilGAP-dependent suppression of cell spreading on collagen (Fig 5A and 5B). In accordance with this result, depletion of endogenous RBM10 by siRNA blocked FilGAP-dependent suppression of cell spreading on collagen (Fig 5C and 5E). Depletion of RBM10 alone did not affect cell spreading (Fig 5E). We generated a construct resistant to RBM10 siRNA KD#1(HA-RBM10^WT-R^) and examined whether down-regulation of FilGAP activity induced by RBM10 siRNA KD#1 was prevented. At 48h post-transfection with RBM10 siRNA KD#1, HA-RBM10^WT-R^, but not wild-type protein, was abundantly expressed in A7 cells (Fig 5F) and A7 cells expressing HA-RBM10^WT-R^ protein rescued the suppression of cell spreading by FilGAP (Fig 5D and 5E). These results suggest that RBM10 may promote suppression of cell spreading by FilGAP downstream of integrin signaling.

### RBM10 is required for Rac GAP activity of FilGAP

**Fig 6:**
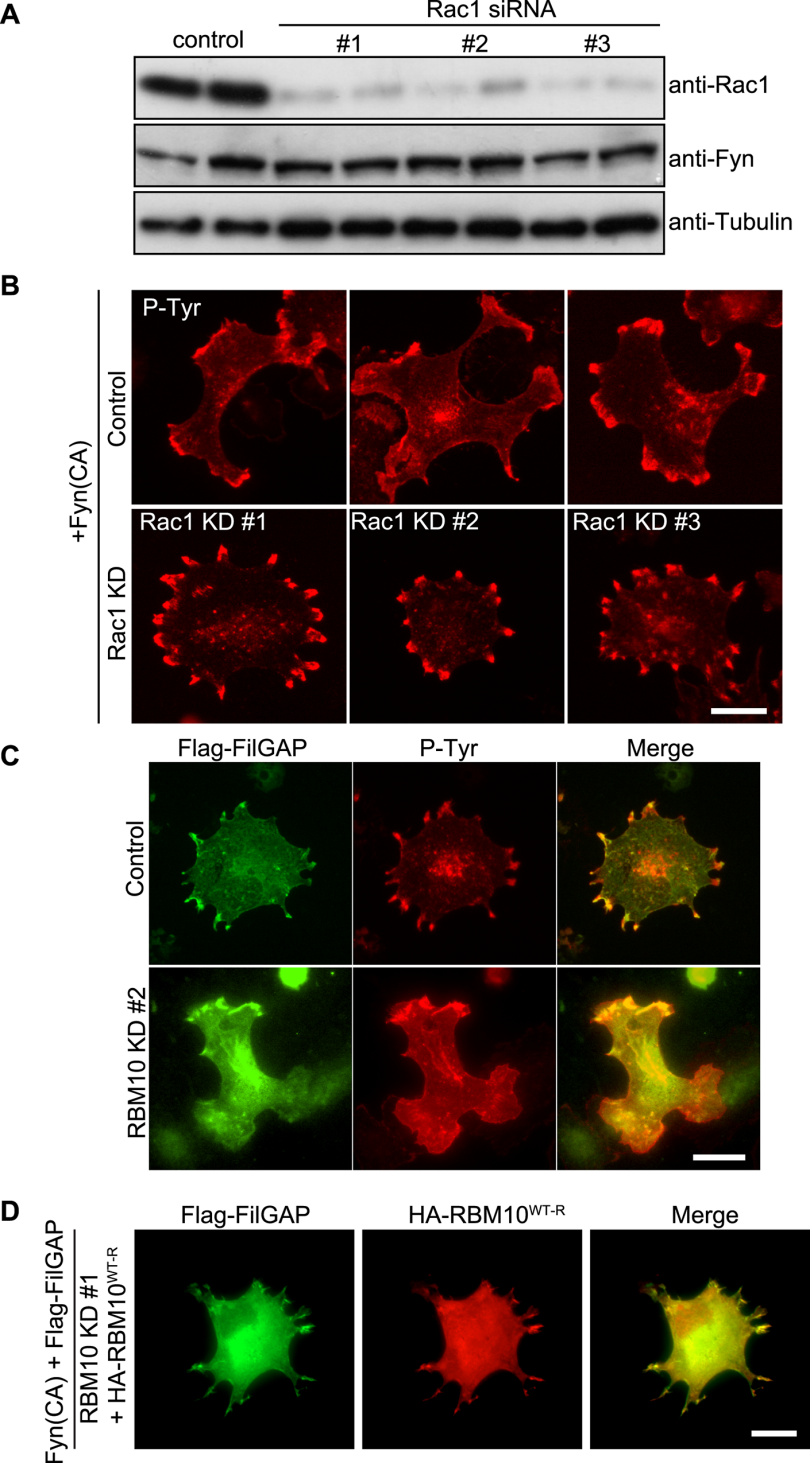
RacGAP activity of FilGAP is stimulated by RBM10. (**A**) Immunoblot showing that Rac1 is depleted after 48 hrs of siRNAs treatment of A7 cells. Tubulin was used as a loading control. (**B**) A7 cells were transiently transfected with constitutively activated Fyn with control or Rac1 siRNAs. After 48 hrs, the cells were fixed and stained with anti-pTyr antibody. Scale bar, 25 μm. (**C**) A7 cells were transiently transfected with CA-Fyn and Flag-FilGAP in the presence of control or RBM10 siRNA. After 48 hrs, the cells were fixed and stained with anti-FilGAP (green) and anti-pTyr (red) antibodies. Merged fluorescent images are also shown. Scale bar, 25 μm. (**D**) A7 cells were transiently transfected with CA-Fyn, Flag-FilGAP, and HA-RBM10^WT-R^ in the presence of RBM10 KD #1 siRNA. After 48hrs, the cells were fixed and stained with anti-FilGAP (green) and anti-HA (red) antibodies. Merged fluorescent images are also shown. Scale bar, 25 μm.

Previous studies have shown that Src family tyrosine kinase Fyn regulates focal complex and lamellipodia [40]. Transfection of CA-Fyn induced lamellae formation, whereas transfection FilGAP suppressed CA-Fyn-induced lamellae formation and instead induced cell protrusions around cell peripheries (Fig 3A and 3B). To examine if these structures are induced by down-regulation of Rac by FilGAP, we studied the effect of knockdown of Rac1 in the presence of CA-Fyn. siRNAs targeting Rac1 reduced the expression of endogenous Rac1 in A7 cells (Fig 6A). Whereas forced expression of CA-Fyn induced large lamellae (Fig 6B), depletion of Rac1 produced small protrusive structures at the cell periphery, which are similar to the structures induced by FilGAP (Fig 6B). In agreement with this result, GAP-deficient FilGAP mutant failed to produce spike structures (S1 Fig). Moreover, over-expression of dominant-negative inhibitor of Rac1 (Rac DN) also induced spike structures (S1 Fig). These results suggest that the formation of small cell protrusions may be due to suppression of Rac activity by FilGAP. Next, to examine if RBM10 is involved in the regulation of FilGAP, we studied the effect of knockdown of RBM10 in A7 cells transfected with CA-Fyn and FilGAP. We found that depletion of RBM10 markedly reduced the formation of small protrusions and instead induced lamellae formation (Fig 6C). Moreover, transfection of siRNA-resistant HA-RBM10 induced cell protrusions at the cell periphery in the presence of RBM10 siRNA (Fig 6D). These results suggest that RacGAP activity of FilGAP might be dependent on RBM10.

### RBM10 promotes FilGAP-dependent suppression of membrane ruffles downstream of EGF signaling

**Fig 7:**
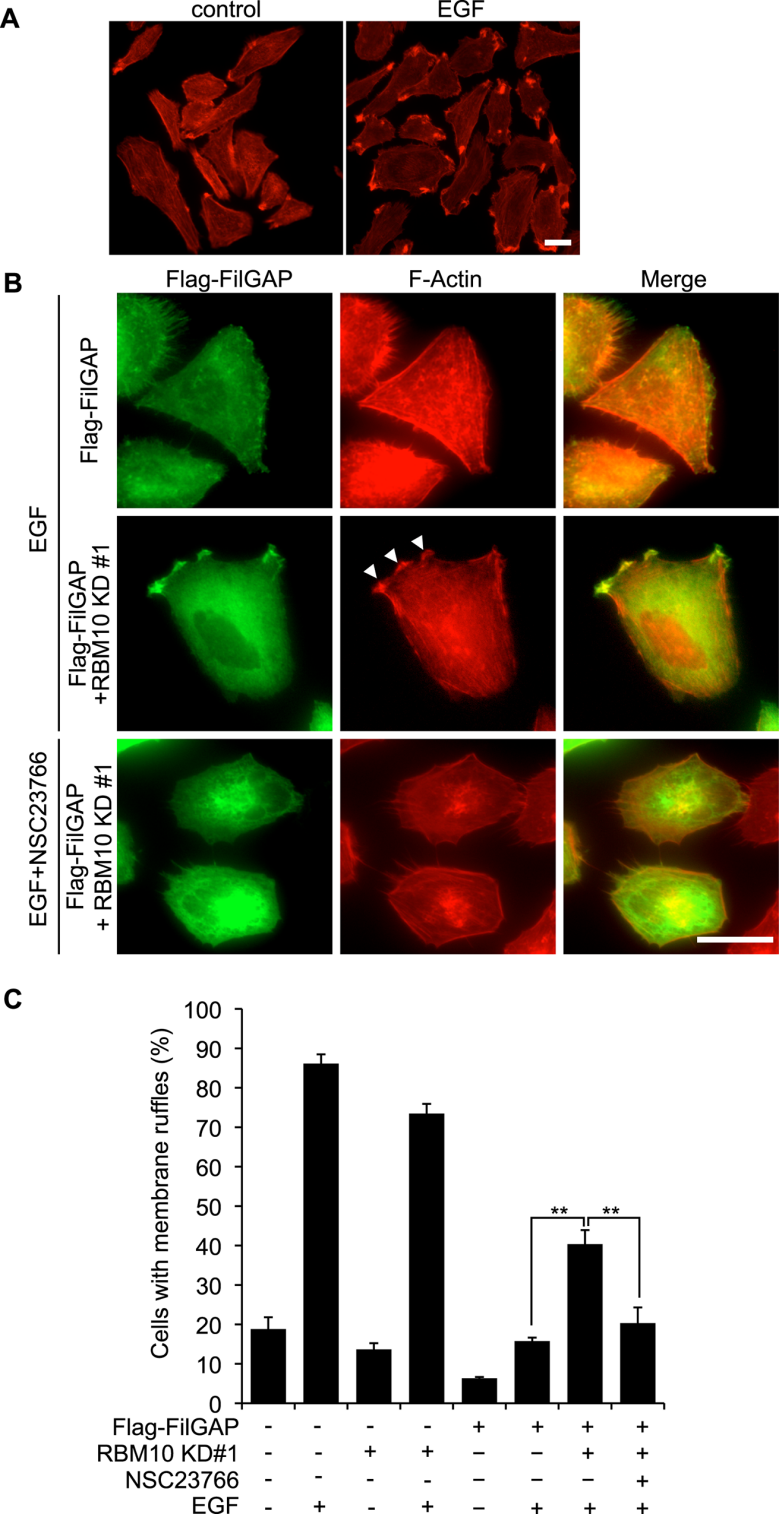
RBM10 promotes FilGAP-dependent suppression of membrane ruffles induced by EGF. (**A**) Serum-starved A7 cells were fixed 30 min after the treatment of the cells with or without EGF (50 nM). Fixed cells were stained with Alexa Fluor568-Phalloidin for F-actin. Scale bar, 25 μm. (**B**) A7 cells were transiently transfected with Flag-FilGAP with control or RBM10 siRNA and serum starved. Cells were pre-incubated with or without 100 μM Rac inhibitor NSC23766 for 1hr and then fixed after the treatment of the cells with 50 nM EGF for 30 min. Fixed cells were stained with anti-FilGAP antibody for FilGAP (green) and Alexa Fluor 568-Phalloidin for F-actin (red). Merged fluorescent images are also shown. Scale bar, 25 μm. Arrowheads indicate membrane ruffles. (**C**) The percentages of membrane ruffling-positive cells were calculated (>300 cells counted for each experiment), and the data are expressed as the mean +/- s.e.m. (n = 3). **, *p*<0.01. The statistical significance was accessed by Student’s *t*-test.

Previous reports indicated that Src family kinases are activated following EGF receptor (EGFR) stimulation [41,42]. Therefore, we examined if RBM10 is involved in FilGAP activities downstream of EGFR. Stimulation of A7 cells with EGF induced the formation of membrane ruffles (Fig 7A and 7C). Transient transfection of A7 cells with FilGAP reduced ruffle formation induced by EGF stimulation (Fig 7B and 7C). Although depletion of RBM10 alone did not stimulate EGF-dependent ruffle formation (Fig 7C), depletion of RBM10 in cells transfected with FilGAP rescued ruffle formation induced by EGF (Fig 7B and 7C). Moreover, ruffle formation induced by knockdown of RBM10 was blocked by Rac1 inhibitor NSC23766 (Fig 7B and 7C). These results suggest that RBM10 may promote FilGAP-dependent suppression of membrane ruffles downstream of EGF signaling through inactivation of Rac.

## Discussion

In this study, we showed RBM10 interacts with FilGAP and provided evidence that Src family tyrosine kinase signaling may control localization and RacGAP activity of FilGAP through association with RBM10.

Our study suggests that RBM10 is required for FilGAP activity downstream of tyrosine kinase signaling. First, forced expression of RBM10 suppressed cell spreading on collagen induced by over-expression of FilGAP. Conversely, depletion of endogenous RBM10 by siRNA blocked FilGAP-mediated suppression of cell spreading on collagen. Moreover, over-expression of siRNA-resistant RBM10 rescued the FilGAP activity. Src/FAK-signaling is required for activation and inactivation of Rho GTPases during integrin-mediated cell spreading on ECM [43]. FilGAP may be regulated downstream of Src/FAK-signaling through association with RBM10. Second, forced expression of CA-Fyn induced lamellae and over-expression of FilGAP suppressed lamellae formation and instead produced small spiky structures. Depletion of RBM10 by siRNA recovered CA-Fyn-mediated lamellae formation in the presence of FilGAP. Formation of spiky protrusive structures appears to be due to inactivation of Rac because the similar structures were also induced by knockdown of endogenous Rac1. Thus, RBM10 seems to be required for FilGAP to suppress Rac downstream of CA-Fyn. Thirdly, over-expression of FilGAP blocked lamellae formation in quiescent A7 cells induced by EGF. Knockdown of endogenous RBM10 produced lamellae in FilGAP-overexpressing cells. The formation of lamellae is dependent on the activation of Rac because Rac-inhibitor NSC23766 blocked lamellae formation. Therefore, RBM10 seems to be required for FilGAP-mediated suppression of Rac downstream of EGF-signaling.

The mechanism of how RBM10 regulates FilGAP activity remains to be determined. However, our result suggests that RBM10 may target FilGAP at focal complexes downstream of SFK-signaling. First, forced expression of CA-Fyn induced translocation of RBM10 from nucleus into cell peripheries, where FilGAP and RBM10 are co-localized. Second, endogenous FilGAP and RBM10 are co-localized with tyrosine-phosphorylated proteins at cell peripheries during cell spreading on collagen. Moreover, depletion of endogenous RBM10 abolished localization of FilGAP at focal complexes. Thus, RBM10 is required for FilGAP to localize at focal complexes.

We showed in this study that the C-terminus of FilGAP interacts mainly with the C-terminus half of RBM10. The interaction does not seem to be mediated by RNA because treatment of cell extract with RNaseA or RNase inhibitor did not abolish the co-precipitation of FilGAP and RBM10. However, the binding does not seem to be a direct interaction because we failed to demonstrate that purified FilGAP protein binds to purified RBM10 protein (data not shown). Moreover, we needed to use cell-permeable cross-linker to show endogenous association between FilGAP and RBM10. Therefore, interaction between FilGAP and RBM10 may be indirect, transient or weak, and they could be a part of large molecular complex and there is FilGAP protein that does not associate with RBM10.

The mechanism of how tyrosine kinase signaling regulates FilGAP also remains to be determined. FilGAP does not seem to be a direct target of Fyn and we did not detect significant tyrosine phosphorylation of FilGAP. We found that RBM10 is phosphorylated at tyrosine residues in CA-Fyn expressing cells. However, CA-Fyn did not increase the binding between FilGAP and RBM10 as determined by co-precipitation experiment (data not shown). We showed that translocation of RBM10 from nucleus into cytoplasm requires Fyn’s kinase activity. Therefore, translocation of RBM10 from nucleus into cell peripheries by CA-Fyn and targeting of FilGAP into focal complex seems to be a major pathway to activate FilGAP as RacGAP. Focal complex is composed of multiple proteins including tyrosine-phosphorylated proteins and SH2- and PTB-domain containing proteins [44]. Focal complex also contains number of actin-binding proteins [37]. Moreover, RNA and RNA-binding proteins have been detected at adhesion sites and shown to be involved in the early stage of cell spreading [45,46]. Further study is required to reveal how SFKs regulate FilGAP activity.

Cell adhesion on ECM induces activation of integrin, which regulates Rho and Rac activities spatially and temporally through activation and inactivation of RhoGAPs and RhoGEFs [31,32]. Our study presents evidence that FilGAP may participate in the inactivation of Rac downstream of integrin-signaling. Integrin-induced lamellae are dynamic structures and they change their morphology during spreading or migration on ECM through protrusion and retraction. Therefore, they may need positive and negative feedback mechanisms to change their behavior. One possible mechanism would be to regulate Rac activity by RacGAPs and RacGEFs simultaneously downstream of tyrosine kinases. Presumably, integrin may target RBM10 to focal complex at the cell periphery, where bound FilGAP may function with other RacGEFs to regulate Rac activity to control lamellae structures during spreading on ECM.

**Fig 8:**
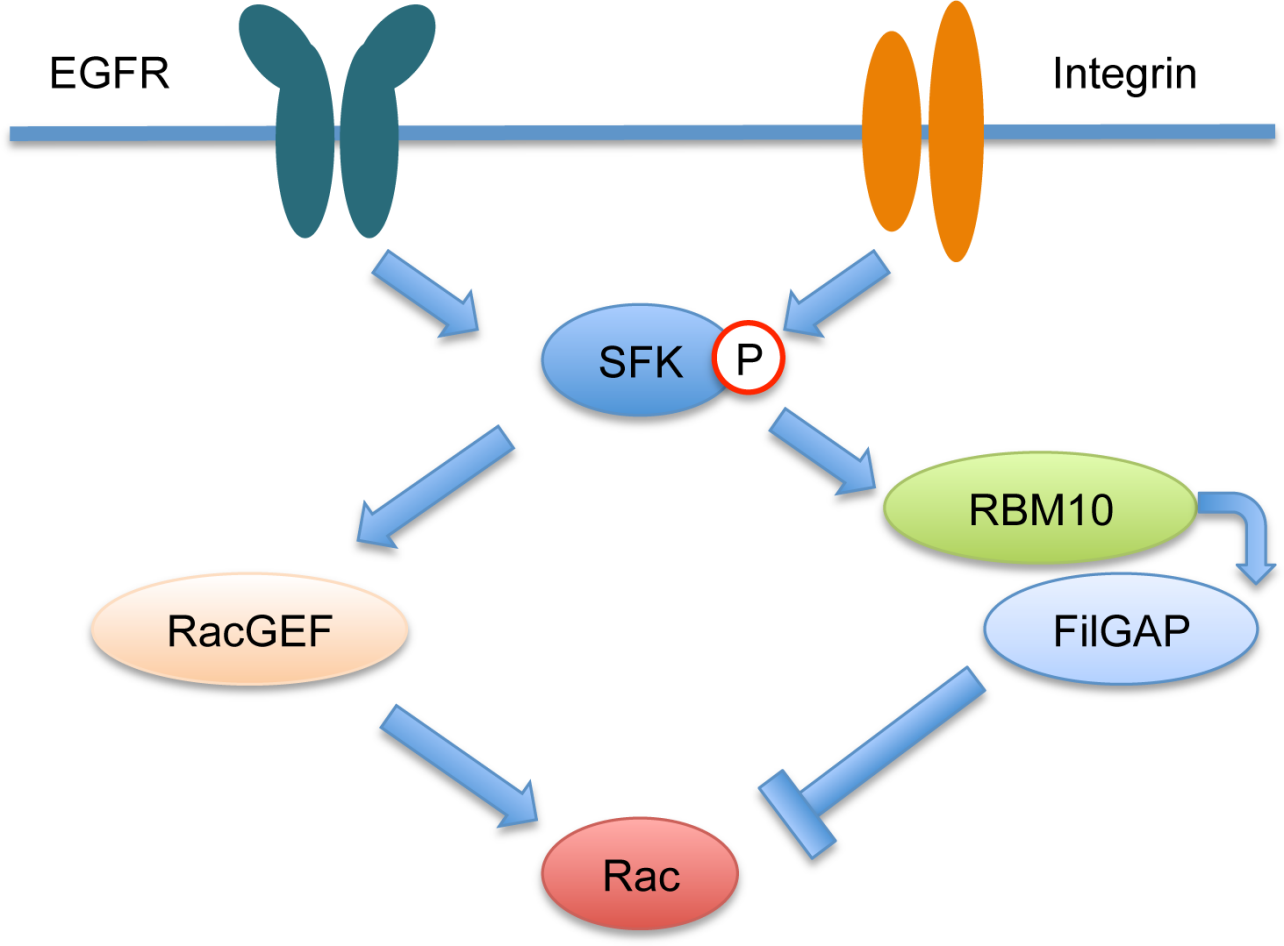
Proposed roles of RBM10 and FilGAP downstream of SFKs signaling to control Rac. Arrows and T-junction represent stimulation and repression.

In summary, we identified RBM10 as FilGAP interacting protein in mammalian cells. Activation of SFKs induced translocation of RBM10 from nucleus into cell peripheries, where FilGAP is targeted into tyrosine-phosphorylated proteins and functions as RacGAP to suppress lamellae formation and cell spreading (Fig 8).

## Materials and Methods

### Plasmids

cDNAs encoding full length *FilGAP* (NM_001025616) and *RBM10* (NP_005667) were described previously [24,28]. cDNAs encoding *RBM10* was obtained from KAZUSA DNA institute (KIAA0122). RBM10 (wild-type, 1–443, 444–930) constructs were generated by PCR and inserted into pCMV5-HA or pCMV5-FLAG vector. FilGAP (1–391) construct in pCMV5-HA was digested with BamH1 and inserted into pCMV5-FLAG vector. FilGAP (552–748) construct in pCMV5-HA was digested with EcoRI and SalI and inserted into pCMV5-FLAG vector. Constitutively active Fyn (Y531F) and kinase negative Fyn (K299M) expression vectors were kindly provided from T. Yamamoto at University of Tokyo.

### Cell culture and transfection

HEK293T cells and human breast carcinoma cell line MDA-MB-231 cell were maintained in Dulbecco’s modified Eagle’s medium supplemented with 10% fetal bovine serum, 50 units/ml penicillin and 50 μg/ml streptomycin at 37°C with 5% CO_2_. Human melanoma cell line A7 cells were maintained in Minimum Essential Medium Eagle supplemented with 8% newborn calf serum, 2% fetal calf serum, 0.5mg/ml G418, 50 units/ml penicillin and 50 μg/ml streptomycin at 37°C with 5% CO_2_. MDA-MB-231 cell line was obtained from the American Type Culture Collection (cat# HTB-26). HEK 293 cell line was obtained from RIKEN Bio Resource Center. A7 cells were provided by Tom Stossel [8]. For transfection, cells were transfected with plasmid DNA using Lipofectamine 2000 (Invitrogen) or siRNA using Lipofectamine RNAimax (Invitrogen) according to the manufacturer’s instructions.

### Identification of FilGAP binding proteins

HEK293T cells were transfected with Flag-FilGAP (amino acid 373–748). Twenty-four hours later, the cells were washed twice in phosphate-buffered saline(PBS) and lysed for 3 min at 27°C with 1ml CSK buffer (0.3 M sucrose, 0.5% TritonX-100, 10 mM PIPES (pH6.8), 100 mM KCl, 1 mM CaCl_2_, 2.5 mM MgCl_2_, 0.1 mM Na_3_VO_4_, 50 mM NaF, Protease Inhibitor Cocktail (SIGMA), and 0.2 mM PMSF). Cell lysates were precleared and precipitates were lysed with RIPA buffer (20 mM Tris/HCl (pH7.5), 120 mM NaCl, 1% Triton-X 100, 0.5% sodium deoxycholate, 0.1% SDS, 10 mM MgCl_2_, Protease Inhibitor Cocktail (SIGMA), and 0.2 mM PMSF). Cell lysates were sonicated and incubated with 15 μl of monoclonal Anti-Flag M2 bead (SIGMA) for 15min at 4°C. The reacted beads were washed 3 times with lysis buffer and 2 times with Tris-buffered saline (TBS). Eluted proteins were separated by SDS-PAGE followed by silver-staining. The band was cut out and digested by trypsin and subjected to LC-MS/MS analysis.

### Immunoprecipitation

HEK293T cells were transiently transfected with Flag-FilGAP and HA-RBM10. After 24 hrs, the cells were washed twice in ice-cold PBS and lysed for 15min on ice with 0.5ml of lysis buffer (50 mM Tris-HCl (pH 8.0), 10% glycerol, 0.1% NP-40, 100 mM NaCl, 2 mM MgCl_2_ 0.2 mM EDTA, 10 mM NaF, 0.2 mM Na_3_VO_4_, Protease inhibitor Cocktail (SIGMA), and 1 mM PMSF). Cell lysates were precleared and supernatant fluid was incubated with 15 μl of monoclonal Anti-HA Agarose beads or Anti-HA affinity gel (SIGMA) for 1hour at 4°C. Immunoprecipitates were washed three times with lysis buffer and bound proteins were detected by SDS-PAGE followed by Western blot. For detection of binding of endogenous FilGAP protein and RBM10 protein, MDA-MB-231 cells were incubated with 1 mM dithiobis (succinimidyl propionate) for 30 min at 25°C. Then, the reaction was stopped by adding 20 mM Tris-HCl (pH 7.4) and incubated for 10 min. The cells were washed, lysed, and cell lysates were precleared and supernatant fluid was subjected to immunoprecipitation with anti-FilGAP antibody to precipitate endogenous FilGAP. Bound protein was detected by western blotting using anti-FilGAP and anti-RBM10 antibodies.

### Immunofluorescence

Cells plated on coverslips were rinsed in PBS and fixed in 3.7% formaldehyde and permeabilized with 0.5% Triton X-100 in PBS at room temperature. Cells were rinsed and incubated with PBS containing 5% horse serum. Cells were incubated with primary antibodies, rinsed in PBS, and further incubated with Alexa Fluor dye-conjugated secondary antibodies. For visualization of F-actin, Alexa Fluor 568-phalloidin was added with secondary antibodies. Cells were rinsed, and coverslips were mounted on slide glass with 10 μl of Aqua-Poly/Mount (Polyscience, Inc.). Cells were examined under fluorescence or phase contrast optics (Olympus). Images were acquired by a charge-coupled device camera (ORCA-ER; Hamamatsu Photonics) and analysed by MetaMorph software (Molecular Devices).

### EGF stimulation

A7 cells were cultured on coverslips (poly-L-lysine coated) transfected with relevant plasmids for 4 h and serum-starved. The cells were fixed after the treatment with 50 ng/ml EGF for 30 min.

### Spreading assay

A7 cells or MDA-MB-231 cells were cultured on 60 mm dish transfected with relevant plasmids for 5 h and serum-starved. After 19 h, the cells were trypsinized and suspended in MEM containing 0.2% BSA. The cells were plated on collagen-coated coverslips (50 μg/cm^2^) and fixed 30 min after plating.

### Antibodies and reagents

Mouse anti-tubulin monoclonal antibody, rabbit anti-HA and anti-FLAG polyclonal antibodies were purchased from Sigma. Mouse anti-HA (12CA5) monoclonal antibody was purchased from Roche Applied Science (Indianapolis, IN). Mouse anti-phosphotyrosine monoclonal antibody (4G10) was purchased from EMD Millipore (Massachusetts, USA). Rabbit anti-Myc polyclonal antibody was purchased from Sigma and MBL (Nagoya, Japan). Rabbit anti-RBM10 polyclonal antibody was purchased from BET (Montgomery, USA). Secondary antibodies conjugated to Alexa Fluor 488 or 568, Alexa Fluor 568-phalloidin were purchased from Invitrogen. Rabbit anti-FilGAP polyclonal antibody was prepared as described previously (Ohta et al., 2006). NSC23766 (Calbiochem) was purchased from EMD Millipore (Massachusetts, USA).

### siRNA

siRNA oligonucleotide duplexes targeting human *RBM10* and *Rac1* were purchased from invitrogen. The targeting sequences were as follows: Rac1, KD#1 5’-UUUACCUACAGCUCCGUCUCCCACC-3’ (nt 24–48), KD#2 5’-AAAUUAAGAACACAUCUGUUUGCGG-3’ (nt 217–241), and KD#3 5’-AGCAAAUUAAGAACACAUCUGUUUG-3’ (nt 220–244); RBM10, KD#1 5’-GAGAACGCCAAUGACACCAUCAUUU-3’ (nt 655–680) and KD#2 5’-CACAAGACAAUGGUGACCCGCUUCA-3’ (nt 2524–2548). For RBM10 siRNA rescue assay, 5 silent mutations were introduced to the siRNA targeting sequence (nucleotides 655–680). The final mutant was changed into GAA^657^AACGCG^663^AATGAT^669^ACCATT^675^ATA^679^T by PCR. The cells were treated with siRNA for 24 h followed by a transfection with rescue constructs. The cells were cultured for another 24 h and proceed for Western blot or spreading assay.

### Statistical analysis

The statistical significance was accessed by two-tailed unpaired Student’s *t*-test or Welch’s *t*-test. Differences were considered to be statistically significant at *p* value of < 0.01. Error bars (s.e.m.) and *p* values were determined from results of at least three experiments.

## Supporting Information

S1 Fig Effect of GAP-deficient FilGAP (FilGAP ∆GAP) and Dominant-negative inhibitor of Rac (Rac DN) on cell morphology induced by CA-Fyn. (**A**) A7 cells were transfected with Flag-tagged wild-type FilGAP (FilGAP WT) or GAP-deficient mutant FilGAP (FilGAP ∆GAP) in the presence of constitutively activated Fyn (CA-Fyn). After 24 h, cells were fixed and Flag-FilGAP (green) and tyrosine-phosphorylated proteins (red) were localized by staining the cells with anti-Flag and anti-pTyr antibodies. Merged fluorescent images are shown. Scale bar, 25 μm. (**B**) A7 cells were transfected with myc-tagged dominant-negative mutant Rac (myc-Rac DN) and constitutively activated Fyn (CA-Fyn). After 24 h, cells were fixed and Flag-FilGAP (green) and tyrosine-phosphorylated proteins (red) were localized by staining the cells with anti-Flag and anti-pTyr antibodies. Merged fluorescent images are shown. Scale bar, 25 μm.
(TIF)
