# Supplementary figures and images for: Src Family Tyrosine Kinase Signaling Regulates FilGAP through Association with RBM10
Source: PLoS One. 2016 Jan 11;11(1):e0146593. doi: 10.1371/journal.pone.0146593 (PMC4709192; doi:10.1371/journal.pone.0146593)

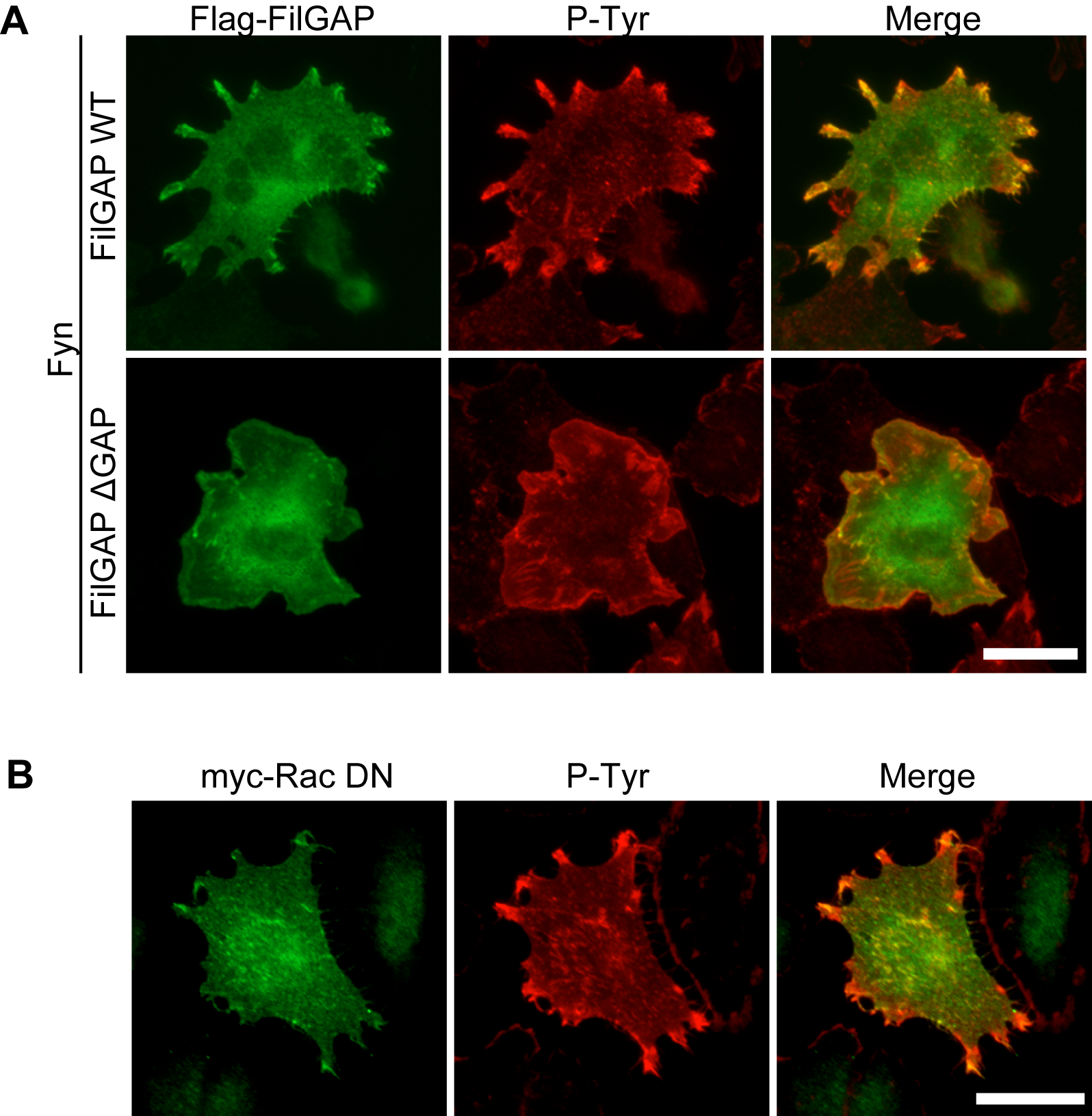

Supplement: S1 Fig — (A) A7 cells were transfected with Flag-tagged wild-type FilGAP (FilGAP WT) or GAP-deficient mutant FilGAP (FilGAP ∆GAP) in the presence of constitutively activated Fyn (CA-Fyn). After 24 h, cells were fixed and Flag-FilGAP (green) and tyrosine-phosphorylated proteins (red) were localized by staining the cells with anti-Flag and anti-pTyr antibodies. Merged fluorescent images are shown. Scale bar, 25 μm. (B) A7 cells were transfected with myc-tagged dominant-negative mutant Rac (myc-Rac DN) and constitutively activated Fyn (CA-Fyn). After 24 h, cells were fixed and Flag-FilGAP (green) and tyrosine-phosphorylated proteins (red) were localized by staining the cells with anti-Flag and anti-pTyr antibodies. Merged fluorescent images are shown. Scale bar, 25 μm. (TIF) [file pone.0146593.s001.tif]
